# Structural studies of the MMP-3 interaction with triple-helical collagen introduce new roles for the enzyme in tissue remodelling

**DOI:** 10.1038/s41598-019-55266-9

**Published:** 2019-12-11

**Authors:** Szymon W. Manka, Dominique Bihan, Richard W. Farndale

**Affiliations:** 1 Nuffield Department of Orthopaedics, Rheumatology and Musculoskeletal Sciences, Kennedy Institute of Rheumatology, University of Oxford, Oxford, UK; 2 Department of Biochemistry, University of Cambridge, Cambridge, UK; 3 Present Address: MRC Prion Unit at UCL, Institute of Prion Diseases, 33 Cleveland Street, London, W1W 7FF UK

**Keywords:** Molecular modelling, Biochemistry, Structural biology

## Abstract

Matrix metalloproteinase-3 (MMP-3) participates in normal extracellular matrix turnover during embryonic development, organ morphogenesis and wound healing, and in tissue-destruction associated with aneurysm, cancer, arthritis and heart failure. Despite its inability to cleave triple-helical collagens, MMP-3 can still bind to them, but the mechanism, location and role of binding are not known. We used the Collagen Toolkits, libraries of triple-helical peptides that embrace the entire helical domains of collagens II and III, to map MMP-3 interaction sites. The enzyme recognises five sites on collagen II and three sites on collagen III. They share a glycine-phenylalanine-hydroxyproline/alanine (GFO/A) motif that is recognised by the enzyme in a context-dependent manner. Neither MMP-3 zymogen (proMMP-3) nor the individual catalytic (Cat) and hemopexin (Hpx) domains of MMP-3 interact with the peptides, revealing cooperative binding of both domains to the triple helix. The Toolkit binding data combined with molecular modelling enabled us to deduce the putative collagen-binding mode of MMP-3, where all three collagen chains make contacts with the enzyme in the valley running across both Cat and Hpx domains. The observed binding pattern casts light on how MMP-3 could regulate collagen turnover and compete with various collagen-binding proteins regulating cell adhesion and proliferation.

## Introduction

Fibrillar collagens I, II and III are the major components of the extracellular matrix (ECM). They provide tissues with tensile strength but also constitute active scaffolds, presenting sites for cell adhesion, for attachment of other ECM components and for storage of many regulatory and signalling molecules^1^. Each collagen consists of three polypeptide α-chains folded into a unique triple helix, with the characteristic Gly-X-Y repeats (where X and Y are often proline and hydroxyproline, respectively) of these chains offset axially by one residue, resulting in distinct leading, middle and trailing chains^2^. Collagens II and III are homotrimers with identical α-chains, whereas collagen I is a heterotrimer comprised of two α1 chains and one α2 chain. These trimers are called tropocollagens and contain an uninterrupted 300 nm long triple helix flanked by short non-helical extensions (telopeptides). At the next level of collagen structure, five tropocollagens assemble side-by-side as a microfibril, with a 64–67 nm (one D-period) axial stagger. One may envisage such blocks organised head-to-tail with a 0.54D gap between them in the collagen fibril, resulting in fibrils and fibres (bundles of fibrils) with the canonical D-periodic cross-striation observed using transmission electron microscopy^3^.

Collagen turnover is slow during homeostasis, but relatively rapid in development, organ morphogenesis, tissue remodelling and wound healing^4^. It is also dramatically unbalanced in fibrotic or degenerative diseases, such as arthritis, atherosclerosis, aneurysm and cancer^5^. Matrix metalloproteinases (MMPs) play major roles in these physiological and pathological processes as they collectively cleave most, if not all ECM components^6^, and can generate paracrine bioactive products^7^. Among them are collagenases: MMP-1, −8 and −13, and stromelysins: MMP-3, −10 and −11, all secreted as inactive zymogens comprising an N-terminal pro-domain and a catalytic domain (Cat) connected to the C-terminal hemopexin domain (Hpx) via a flexible linker (hinge region). The main difference between these two enzyme sub-groups is that stromelysins do not cleave the triple-helical regions of fibrillar collagens, despite sharing a similar degree of structural identity amongst themselves and with the collagenases (>50% sequence identity and virtually superposable tertiary structures of their Cat and Hpx domains^6,8^. Although MMP-3 is not directly collagenolytic, it is a critical procollagenase activator^9,10^. The prototypic collagenase MMP-1 has only 10–20% of its maximal collagenolytic activity without proteolytic activation by MMP-3^9^. Hence, proMMP-3 is often secreted together with proMMP-1 by mesenchymal cells, macrophages and cancer cells stimulated with pro-inflammatory cytokines^7^.

Besides activating procollagenases and other proMMPs, MMP-3 itself breaks down multiple ECM components, including proteoglycans, fibronectin, laminin, collagen telopeptides and basal lamina collagen IV; cell surface proteins, such as E-cadherin at cell junctions; and other non-ECM molecules influencing cell proliferation and differentiation^11^. The enzyme is upregulated in many diseases. For example, after myocardial infarction it may serve as a predictor of adverse left ventricular remodelling and dysfunction^12^ and in idiopathic pulmonary fibrosis it may disrupt lung epithelia through cleavage of E-cadherin^13^, causing epithelial-to-mesenchymal transition. MMP-3 is also found in involuting mammary gland, cycling endometrium, long bone growth plate, atherosclerotic plaque, gastrointestinal ulcers, around various tumours and in other tissue remodelling contexts, reviewed by Nagase^11^. MMP-3 is thus considered an upstream regulator of tissue remodelling in health and disease^14^, but its exact *in vivo* roles are not clearly established. For example, in rheumatoid arthritis, MMP-3 is abundantly expressed in cartilage (reaching ~2 μM concentration)^15,16^ and serum levels are used in diagnostics, but MMP-3 knockouts give conflicting outcomes in different disease models^17–19^.

Although MMP-3 does not cleave triple-helical regions of fibrillar collagens, it can bind to them^20,21^, which may be the basis of its tissue retention and which may directly or indirectly impact collagen and non-collagenous matrix turnover. Here, we report the locations of MMP-3 binding sites along tropocollagens II and III using the Collagen Toolkits that proved useful in mapping the footprints of MMP-1^22^ and MMP-13^23^ on collagens II and III, and those of many other collagen-binding proteins^24,25^. The Toolkit screening reveals that MMP-3 binding to triple-helical collagen requires a Phe residue in position X of the Gly-X-Y repeat, and that the recognition of this critical motif relies on cooperative binding of both Cat and Hpx domains. Having combined computational methods with experimental restraints, we propose an integrative model of an MMP-3-collagen complex. We show that the multi-site binding of MMP-3 to fibril-forming collagens can influence their fibrillogenesis, which *in vivo* may alter their exposure to collagenases, providing an additional mechanism of regulation of collagen degradation. Lastly, we discuss the potential consequences of MMP-3 binding to collagens II and III with respect to other binding partners of these collagens.

## Results

### Both cat and Hpx domains of MMP-3 participate in the binding of the triple helix

**Figure 1:**
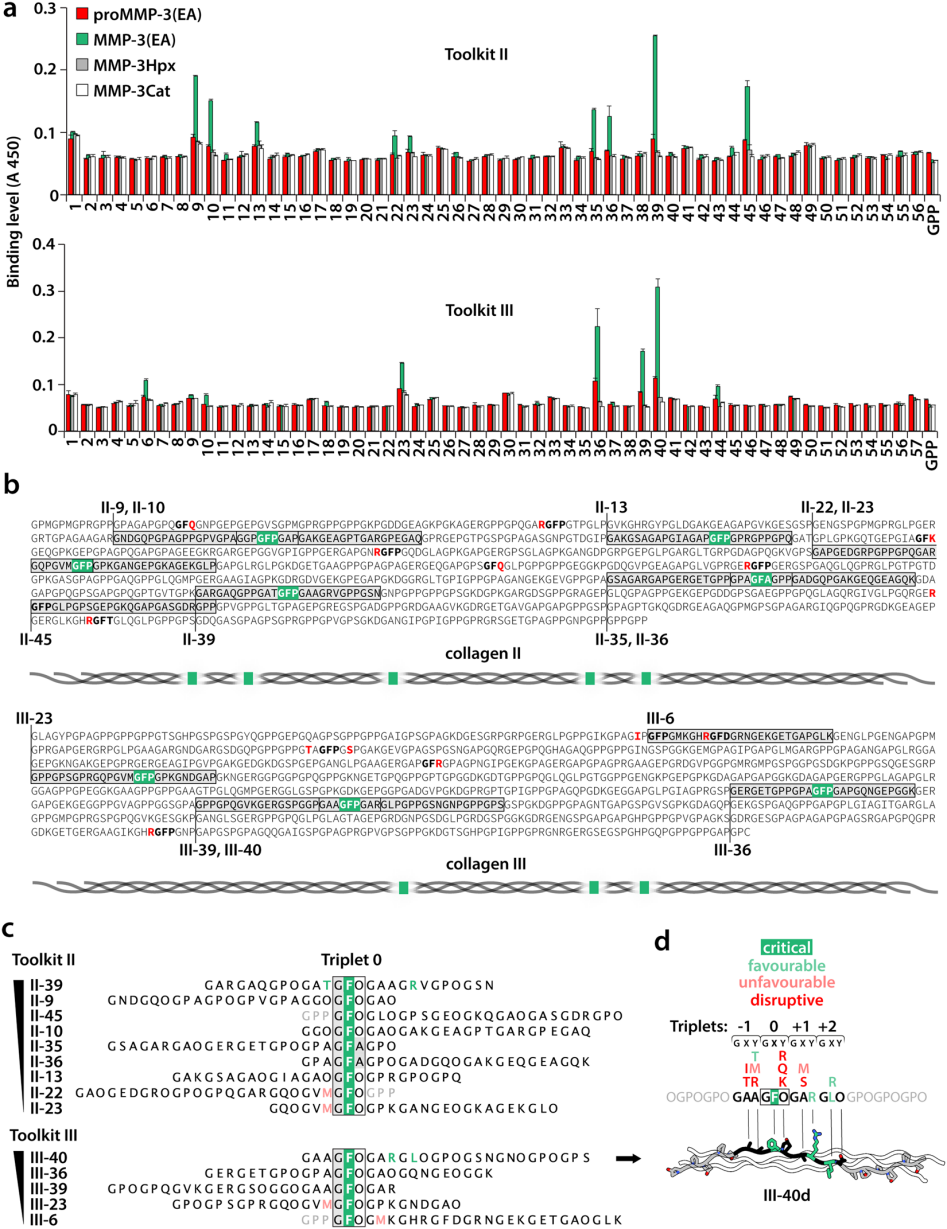
(**a**) Relative binding of biotinylated MMP-3 constructs to Toolkits II and III. The results are the average of 3 independent binding assays performed at room temperature (~20 °C). Error bars are standard deviations (SD). GPP: a THP with repeating Gly-Pro-Pro sequence. (**b**) Analysis of collagen sequence determinants for MMP-3 binding based on the Toolkit data shown in **a**. MMP-3-bound peptides are located in the triple-helical domains of human collagens II and III (Uniprot identifiers: P02458 and P02461, respectively). The sequences do not include post-translational modification of proline (P) at the position Y of the GXY repeat to hydroxyproline (O). MMP-3 binds GFO/A (here GFP/A)-containing sites, unless they are preceded or followed by residues marked in red. The critical GFP/A triplet is highlighted in green wherever it mediates MMP-3 binding. Included are schematic overviews of the MMP-3 binding site distributions along tropocollagens II and III. The GFP site represented by peptides II-44/45 is not expected to be recognised by MMP-3. Peptide II-45, starting from the critical GFO triplet and therefore lacking the preceding disruptive R residue, is bound, whilst the overlapping peptide II-44 is not. (**c**) Sorting the MMP-3-bound peptides according to relative MMP-3 binding affinity. Peptides are aligned on the critical GFO/A triplet designated Triplet 0. One triplet of the *host* sequence is included (grey font) for peptides starting or ending with Triplet 0. Residues that appear as advantageous for the binding are coloured green and those that appear unfavourable are coloured pale red. (**d**) Summary of favourable and unfavourable sequence motifs for MMP-3 binding shown together with a computationally modelled triple-helical peptide (THP) III-40d, designed for modelling of the MMP-3:THP complex. The THP is based on the optimal MMP-3 recognition sequence identified in peptide III-40 (with highest apparent MMP-3 affinity of all Toolkit peptides), consisting of 4 triplets designated: −1, 0, +1, +2. These and the flanking GPO triplets were generated by *mutating* collagen III peptide 1BKV^26^ in *Coot*^74^. For clarity, only one THP chain is shown with coloured residue side chains. Element colours: N, navy blue; O, red.

Toolkit II contains 56 triple-helical peptides (THP) and Toolkit III, 57. Every THP in each Toolkit contains 27 amino acids (aa) of the respective collagen (guest) sequence, flanked by 5 GPP repeats and a GPC triplet (host sequence). The first and the last 9 aa of the guest sequence overlap with the preceding and the consecutive THP in the series, respectively. We used a solid phase binding assay to screen these peptide libraries for binding of biotinylated proMMP-3(E200A), mature MMP-3(E200A) and the isolated Cat and Hpx domains. The active-site mutation (E200A) does not affect the conformation of MMP-3 but prevents autolysis and autoactivation. ProMMP-3(E200A) and the Cat and Hpx domains alone showed no prominent binding to any of the Toolkit peptides (Fig. 1a). Only the mature MMP-3(E200A) specifically recognised 9 THPs in Toolkit II: 9, 10, 13, 22, 23, 35, 36, 39 and 45 (A_450_ ≥ 0.1, except peptide 1, where the signal does not appear specific due to the high background), and 5 THPs in Toolkit III: 6, 23, 36, 39, 40 (A_450_ > 0.1). THP III-44, which contains the canonical collagenase cleavage site, may also be considered to have marginal affinity for MMP-3. Some THPs, like III-36 and III-40, seem to weakly interact with proMMP-3(E200A), but this binding appears negligible compared to the activated MMP-3(E200A) (Fig. 1a).

### MMP-3 recognises triple helices encompassing GFO/A triplets in particular contexts

All the peptides that firmly interacted with MMP-3(E200A) have one unifying feature, the GFO/A triplet, which we designated Triplet 0 (Fig. 1b,c). Notably, Phe at position X is not recognised by MMP-3(E200A) when accompanied by Arg, Gln or Lys at position Y (Fig. 1b,d).

Binding to Triplet 0 depends on a wider sequence context, since it is found at 4 and 3 sites on collagens II and III, respectively (Fig. 1b), that are not recognised by MMP-3(E200A) according to the Toolkit screening. In these cases, the Triplet 0 occurs in the following contexts: R-GFO, IP-GFO or TA-GFO-GS. Therefore, Arg directly preceding the Triplet 0, Ile or Thr at position X of the preceding triplet (Triplet −1) and/or Ser at position X of the following triplet (Triplet +1) appear to disrupt MMP-3(E200A) binding (Fig. 1b,d). This implies that THPs II-45 and III-6 are recognised only because their guest sequences start from Triplet 0 and, therefore, exclude the disruptive residues that precede them in native collagens (Fig. 1b). This is supported by the clear lack of recognition of the neighbouring THPs II-44 and III-5 that overlap the sites in question (Fig. 1a). We thus conclude that THPs II-45 and III-6 do not represent true binding sites of MMP-3 on native tropocollagens. Consequently, there are 5 and 3 unique interaction sites for MMP-3 on tropocollagens II and III, respectively (Fig. 1b).

To identify the more and less favourable residues for MMP-3 binding in the vicinity of Triplet 0 we ranked the MMP-3(E200A)-binding THPs according to their relative affinity to MMP-3(E200A) (Fig. 1c). Our analysis indicates that Met at position Y of the Triplet −1 or at position X of the Triplet +1 is unfavourable, while Thr at position Y of the Triplet −1, Arg at position Y of the Triplet +1 and Leu or Arg at position X of the Triplet +2 are favourable for MMP-3 binding (Fig. 1c,d).

### Integrative modelling of the MMP-3:THP complex

We predict modes of MMP-3 binding to triple-helical collagen by integrating the established THP binding specificity with the mechanistic insights derived from the Toolkit screening: i) that the pro-domain of MMP-3 zymogen interferes with THP binding, and ii) that both the Cat and Hpx domains of MMP-3 are required for binding. The sequence of the most strongly bound THP III-40 served to generate a computational model THP, III-40d, for optimal molecular docking to an MMP-3 model (Fig. 1d). III-40d contains 5 triplets (−1 to +3) of the THP III-40 and 2 flanking GPO repeats built on the backbone of an X-ray THP structure 1BKV.pdb^26^ (Fig. 1d).

To fully explore the experimental restraints for the subsequent docking experiment, we modelled both the pro-form and the mature form of MMP-3, in the absence of experimental structures. ProMMP-3 homology modelling was based on proMMP-1 crystal structure (1SU3.pdb^27^), and the full-length mature MMP-3 was based on several homologous structures available: active MMP-1 (2CLT.pdb^28^), MMP-1(E200A) in a complex with a THP (4AUO.pdb^22^) and two structures of MMP-13 (4FU4.pdb and 4FVL.pdb^29^). All models were obtained with Modeller^30^ and were of highest quality, reaching maximal GA341 scores = 1.0 (native-like structures)^31,32^. The best structure in each collection of models was selected according to the lowest Discrete Optimized Protein Energy (DOPE) scores^33^ (see details in Supplementary Fig. S1).

**Figure 2:**
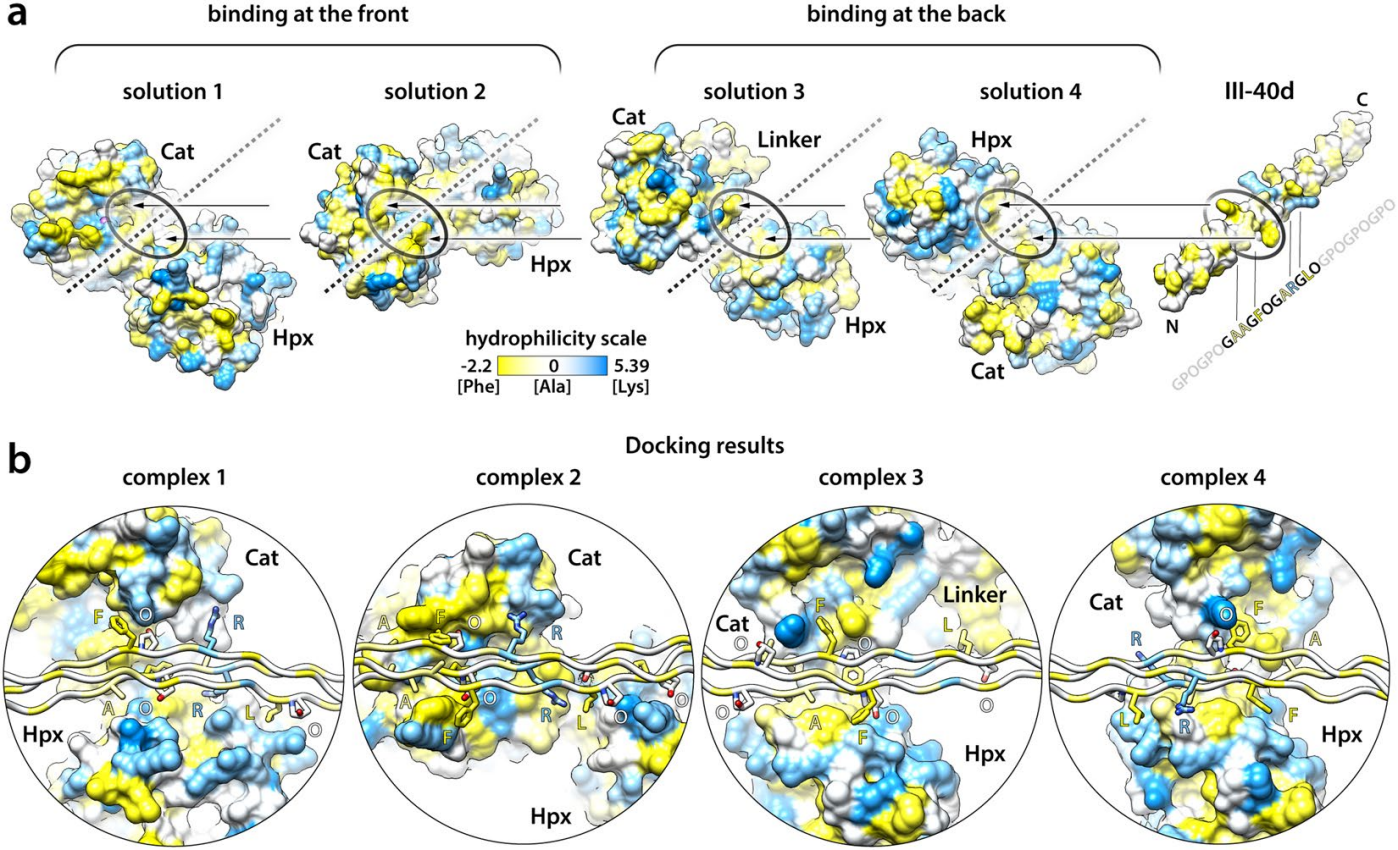
(**a**) MMP-3 and III-40d THP models represented with solvent-accessible surfaces (SAS) (UCSF Chimera^75^). Colour is according to hydrophilicity^34^ and values range from −2.2 for Phe (most hydrophobic, yellow) to 5.39 for Lys (most hydrophilic). Four hydrophobic patches are indicated with oval shapes on the MMP-3 surface. Each can accommodate the critical GFO/A triplet of the THP in a way that would involve both the Cat and Hpx domains in its binding: two sites are at the front, and two at the back of MMP-3, according to the standard MMP-3 orientation shown in the Supplementary Fig. S1. The orientation of MMP-3 in each collagen binding solution (1–4) is shown relative to the fixed axis of the III-40d THP, indicated with broken lines. For subsequent refinement of each MMP-3:III-40d binding interface with Rosetta^35^, the THP has been roughly positioned ~5 Å from the MMP-3 homology model, as indicated by the arrows. (**b**) Results of the local docking and interface refinement by Rosetta^35^. Complexes 1–4 correspond to the putative THP/collagen binding solutions 1–4 shown in (**a**). The III-40d THP is oriented horizontally for each model, demonstrating various degrees of THP bending in the modelled complexes. MMP-3 is shown with SAS (as in **a**) and the THP as ribbon, with side chains of the residues containing at least one atom located within 4 Å distance from MMP-3 shown as sticks and labelled with one-letter codes; colouring as in (**a**) and by heteroatom: N, navy blue; O, red. The hydrophilicity colouring algorithm works on a per residue basis, i.e. colours the whole residue according to the assigned value. Thus, hydrophobicity of the aliphatic stalks of the side chains such as Lys or Arg is not accounted for.

Since Phe in the Triplet 0 is central to MMP-3 binding, the complex formation must be driven by a hydrophobic interaction. We visualised hydrophobic patches on the surface of the MMP-3 model using hydrophilicity scale proposed by Moon and Fleming^34^, with values ranging from −2.2 for Phe (most hydrophobic) to 5.39 for Lys (most hydrophilic) (Fig. 2). We considered the hydrophobic regions that: i) can accommodate a triple helix, ii) involve both Cat and Hpx domains in the resultant THP binding mode, and iii) are sensitive to the presence of the pro-domain. We identified four candidate solutions that would satisfy all these experimentally derived criteria: two pertain to collagen binding at the front, and two at the back of the enzyme (Fig. 2a). The two frontal-binding possibilities (solutions 1 and 2) would be directly blocked by the pro-domain, whereas the two rear-binding possibilities (solutions 3 and 4) were also considered, since the relative positions of the Cat and Hpx domains could be altered allosterically by the pro-domain, perturbing the collagen binding site (Supplementary Fig. S2). The THP model III-40d was docked to the MMP-3 model in the four proposed ways (Fig. 2a), yielding excellent complexes after Rosetta refinement^35^ in all cases (Fig. 2b and Supplementary Table S1). Each complex bends the triple helix somewhat, especially complex 1, this however does not affect the geometry of the THP (Supplementary Table S1).

### Complex 2 represents the putative collagen binding mode of MMP-3

**Figure 3:**
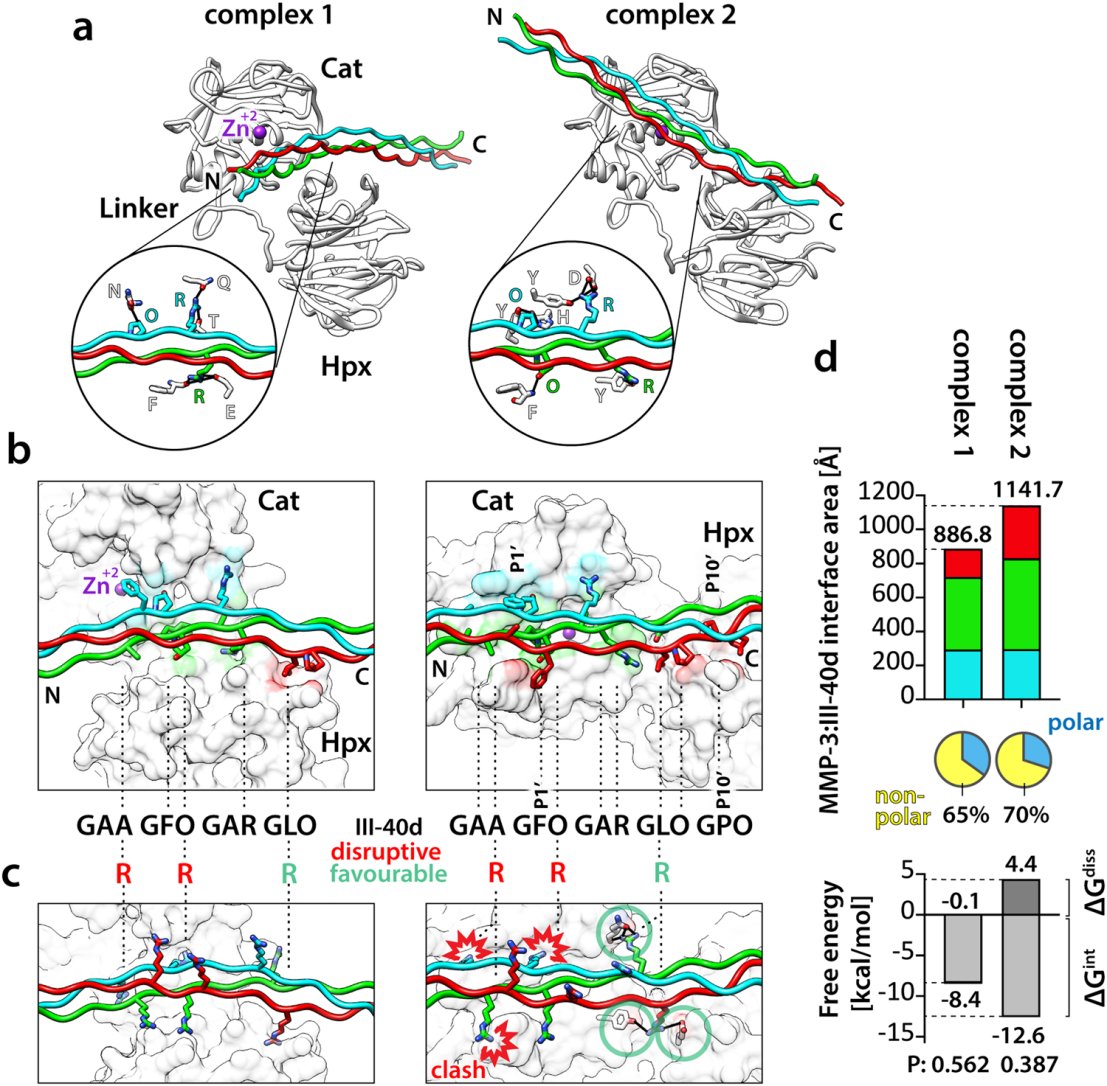
(**a**) Two modelled frontal-binding MMP-3:III-40d complexes shown as ribbons in the standard MMP-3 orientation. MMP-3 chain, white; Zn^2+^, magenta. THP chains: leading, blue; middle, green; trailing, red. Circles, close-up views of MMP-3:III-40d hydrogen bond networks (black lines), with the bonding residues represented as sticks coloured by heteroatom: N, navy blue; O, red, and labelled with 1-letter codes. (**b**) Close-up view of the modelled MMP-3:III-40d interfaces. The THP is represented as in (**a**), with side chains containing at least one atom located within 4 Å of the MMP-3 shown as sticks coloured by heteroatom: N, navy blue; O, red. MMP-3 is shown as solvent-accessible surface (UCSF Chimera^75^) coloured according to the interfacing THP chains (4 Å distance). IDs of the staggered residues in the III-40d THP are roughly indicated (dashed lines). The P1’ and P10’ sites (nomenclature^37^) are indicated for complex 2. In each complex, all three chains of the THP make contacts with MMP-3. (**c**) Theoretical analysis of the effects of III-40d residue substitutions on MMP-3 binding. MMP-3 is shown as white and transparent SAS and III-40d is shown as in (**a)**, with modelled Arg side chains and hydrogen-bonded MMP-3 residues represented with sticks coloured by heteroatom: N, navy blue; O, red. Arg at the position Y of the Triplets −1 and 0 causes severe clashes with MMP-3 only in complex 2. (**d**) Top, NACCESS^36^ computation of the modelled MMP-3:III-40d interface areas, colour-coded as in (**b)**. Pie charts indicate the polar and nonpolar fractions of the total interface area for each modelled complex. Bottom, PDBePISA computation of the MMP-3:III-40d binding energies in each modelled complex. ΔG^int^, estimated solvation free energy gain upon complex formation, excluding the effect of satisfied hydrogen bonds and salt bridges across the interfaces. ΔG^int^ < 0 corresponds to hydrophobic interfaces, or positive protein affinity. The ΔG^int^ P-value indicates interface specificity: P = 0.5, not specific; P > 0.5, likely an artefact; P < 0.5, likely interaction-specific. ΔG^diss^, estimated free energy needed to dissociate the assembly; interfaces with ΔG^diss^ > 0 are thermodynamically stable.

Figure 3a shows the two frontal-binding candidate MMP-3:THP complexes 1 and 2 in the standard enzyme orientation. The rear-binding complexes 3 and 4 are shown with equivalent representations in Supplementary Fig. S3. All three α-chains make contacts with MMP-3 in all complexes (Fig. 3b and Supplementary Fig. S3). In theory, the enzyme could be using several modes of collagen binding with variable preference for particular sites, and all four candidate complexes seem legitimate. Without experimental data it would be impossible to rank them in terms of the likelihood of their representing the true mechanism. However, the Toolkit screening has endowed us with knowledge of residues that are favourable and disruptive for MMP-3 binding at defined THP positions (Fig. 1d). So, we set out to validate each candidate complex by substituting selected residues in the THP model III-40d for those that showed most profound effects on MMP-3 binding. To test the expected disruptive effects, we swapped Ala and Hyp at positions Y of the Triplets −1 and 0, respectively, to Arg (Fig. 3c and Supplementary Fig. S3), which cancels MMP-3(E200A) binding (Fig. 1b,d). Only complex 2 showed severe clashes between the MMP-3 model and the Arg side chains and no obvious clashes arose in complexes 1, 3 and 4 (Fig. 3c and Supplementary Fig. S3). For completeness, we also tested the expected beneficial effects of swapping Leu at position X of the Triplet +2 to Arg (Fig. 1c,d). Complex 1 showed no change upon this substitution (Fig. 3c), complexes 3 and 4 each showed a single additional hydrogen bond (Supplementary Fig. S3), and complex 2 showed four extra hydrogen bonds (Fig. 3c). It turns out that only complex 2 is completely in line with all our experimental evidence. We therefore conclude that complex 2 represents the best putative mode of collagen binding by MMP-3 and that the other three candidate complexes are probably modelling artefacts (so-called decoys).

This is further supported by computational analyses of the interfaces performed with NACCESS^36^ and the PDBePISA server (http://www.ebi.ac.uk/msd-srv/prot_int/cgi-bin/piserver). The total MMP-3:THP interface area is largest in complex 2 (1141.7 Å^2^) of which 70% constitutes a hydrophobic interface (Fig. 3d). Moreover, it exhibits thermodynamically favourable parameters, such as the ΔG^int^ P-value of 0.387 (probability of getting a lower than observed solvation free energy gain upon complex formation, when the interface atoms are picked randomly from the protein surface, until the observed interface area is achieved) and the ΔG^diss^ value of 4.4 kcal/mol (external driving force required to dissociate the assembly), which indicate a specific and thermodynamically stable interaction (Fig. 3d).

### MMP-3 cleaves only unfolding collagen chains including those pre-cleaved by collagenase

**Figure 4:**
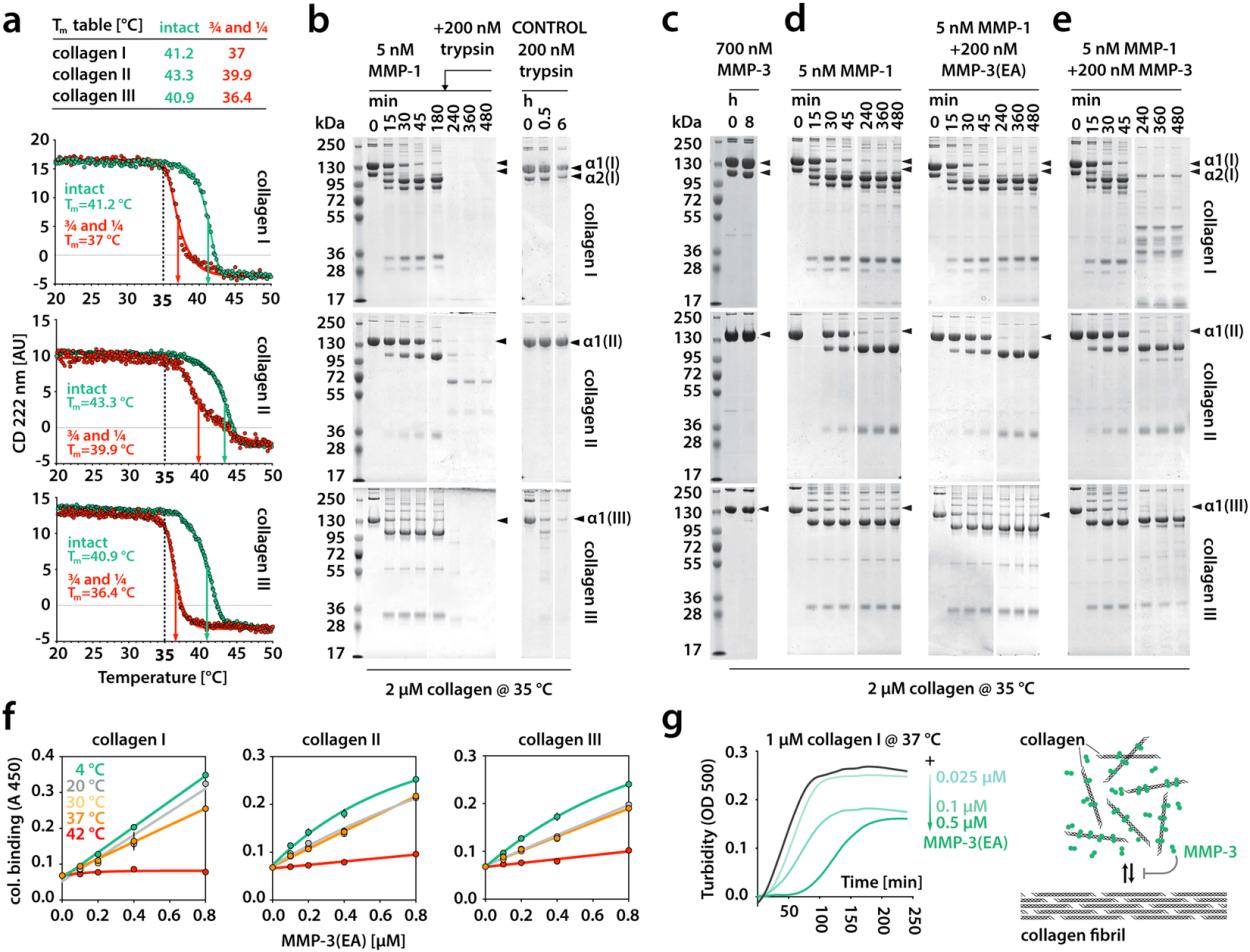
(**a**) Circular dichroism (CD) analysis of the melting temperatures (T_m_) of native fibrillar collagens and their MMP-1 cleavage products (¾ and ¼ fragments). (**b–e**) SDS-PAGE analyses of various collagen digestion experiments. (**f**) Temperature-dependent binding of biotinylated MMP-3(EA) to collagens I, II and III. The results are the average of an assay in triplicate. Error bars (SD) are smaller than the data point where not visible. (**g**) Left, collagen fibrillogenesis at increasing concentrations of MMP-3(EA), measured by turbidity at 500 nm wavelength (OD 500). Right, schematic illustration of collagen fibrillogenesis impairment caused by MMP-3 binding to tropocollagen molecules. MMP-3(EA) denotes the active site mutant of MMP-3.

The established putative mode of collagen binding by non-collagenolytic MMP-3 appears similar to that determined for collagenase MMP-1^22^, but their collagen-binding specificities are completely different. MMP-1 binding relies on Leu residues at P1’ and P10’ positions (nomenclature after Schechter and Berger^37^) of collagen. The P1’Leu is bound by MMP-1 Cat domain and the P10’Leu is bound by MMP-1 Hpx domain (so-called *exosite*-binding), and both interactions are critical for collagenolysis^22^. Although MMP-3 also appears to require its Hpx domain for collagen binding, it does not show a strong preference for any specific residue at the site equivalent to the P10’ site of collagen (position X of the Triplet 3: Pro in the THP III-40d) (Fig. 3b). MMP-3 only requires Phe residue at the site equivalent to the P1’ site of collagen (position X of the Triple 0), to bind to collagen, so we wondered what the biological role of MMP-3 binding to collagen could be. First, we investigated whether MMP-3 can directly influence collagen degradation at any stage of the process, and for that we used native tropocollagens I, II and III devoid of telopeptides to prevent fibril formation. In order to pinpoint the stage of collagen breakdown at which MMP-3 may be relevant, we needed to establish the temperature at which our collagen samples and their collagenase cleavage products (the ¾ and ¼ fragments) lose triple-helicity (T_m_, melting point). The thermal stability of tropocollagens and their fragments after digestion with MMP-1 was measured using circular dichroism (Fig. 4a). The collagen II helix was most thermostable (T_m_ = 43.3 °C) and collagen III the least (T_m_ = 40.9 °C). Likewise, the ¾ and ¼ fragments of collagen II were most thermostable (T_m_ = 39.9 °C) and those of collagen III the least (T_m_ = 36.4 °C). The triple helix is normally resistant to trypsin, which can thus be used as a reporter of collagen triple-helicity. Our trypsin digestion test revealed local thermal instability of the collagen III fold (sensitivity to trypsin) at 35 °C (Fig. 4b), which is below the threshold of global collagen III unfolding (Fig. 4a). Both collagens I and II were resistant to trypsin at 35 °C, but the MMP-1-cleaved products of all the collagens were readily cleaved by trypsin at this temperature (Fig. 4b), which indicates that the clipped ends already start to unfold at 35 °C. This is apparent in the melting curves of the collagen I and III fragments, but not in that of collagen II fragments. Consistently, collagen II fragments showed the lowest susceptibility to trypsin at 35 °C (Fig. 4b). We confirmed that at 35 °C MMP-3 was in principle unable to cleave the native triple helix, although collagen III appeared marginally cleaved approximately at the collagenase cleavage site after 8 h (Fig. 4c). This weak activity against collagen III has been reported^38^ and is consistent with its local thermal lability (breathing) at 35 °C (Fig. 4b) and with the marginal binding of MMP-3(E200A) to THP III-44 (Fig. 1a), where the collagenase cleavage site resides. To test if MMP-3 binding to collagen has any effect on collagenolysis by MMP-1, we compared the collagenolytic activity of 5 nM MMP-1 in the presence or absence of proteolytically inactive 200 nM MMP-3(E200A). The binding of MMP-3(E200A) to collagen did not interfere with collagenolysis by MMP-1 (Fig. 4d). Finally, we tested MMP-3 activity at 35 °C on the labile ¾ and ¼ fragments of collagens I, II and III generated by MMP-1. The fragments of the heterotrimeric collagen I (two α1(I) chains and one α2(I) chain) were relatively readily cleaved by 200 nM MMP-3, especially the α2(I) chain, while those of homotrimeric collagens II and III were poorly cleaved (Fig. 4e), even though collagen III fragments were least thermostable (Fig. 4a). Overall, MMP-3 shows gelatinase activity, as the collagen fragments that begin to melt at 35 °C (Fig. 4b) are equivalent of gelatin (denatured collagen) for the enzyme.

### MMP-3 affects collagen fibrillogenesis *in vitro*

To test the importance of the triple-helicity of collagen for MMP-3 binding we performed the solid phase binding assay at various temperatures using collagens I, II and III as substrates (Fig. 4f). In line with our molecular modelling (Fig. 3), collagen binding to MMP-3(E200A) is highly dependent on the triple-helical structure (Fig. 4f): the lower the temperature, the higher the binding. This may explain the overall weak proteolytic activity of MMP-3 on the gelatinised (unfolding) ¾ and ¼ collagenase cleavage products at 35 °C (Fig. 4f). We then used a collagen I sample containing telopeptides to test whether MMP-3 binding to such a fibril-forming collagen may interfere with fibril formation (Fig. 4g). As expected from the number of MMP-3 binding sites along tropocollagens II and III, MMP-3(E200A) dose-dependently slowed down collagen I fibrillogenesis at 37 °C, as measured by turbidimetry at 500 nm wavelength (Fig. 4g). This suggests that one of the direct functions of MMP-3 binding to the collagen triple helix could be to regulate collagen fibrillogenesis.

## Discussion

The many overlapping activities and biochemical properties of MMPs, and their complex pleiotropic effects under various physiological or pathological circumstances, render it extremely difficult to unravel the exact role of any particular MMP. To approach this question, structural insights are helpful, as every mechanistic clue offers a leap in understanding of the functional potential. Here we focused on MMP-3 (stromelysin 1) which, like other MMPs, can mediate a vast number of events in the body. Its prolonged presence in tissues via immobilisation on collagen, the most abundant protein in the body, would be expected to have profound consequences. The goal of this study was to gain a better understanding of these potential consequences through the elucidation of structural details of the MMP-3 interaction with the interstitial fibril-forming collagens.

The identification of multiple MMP-3 binding sites on collagens II and III, each exhibiting different affinity for MMP-3, explains the apparently unsaturable collagen binding by MMP- 3 reported previously^21^ and also seen here (Fig. 4f). These binding sites are distinct from those previously identified with the Toolkit screening for the collagenases MMP-1^22^ and MMP-13^23^. Despite its marked homology with these collagenases, MMP-3 fails to recognise the unique site on interstitial collagens to which they bind, which may explain why MMP-3 is not collagenolytic. The triple helix binding mechanism relies on different structural features in these enzymes, although for both MMP-1 and MMP-3 it is generally driven by hydrophobic interactions and involves both Cat and Hpx domains^22^.

An important difference between the collagenolytic MMP-1 and non-collagenolytic MMP-3 is that MMP-1 prefers a loose triple helix at 25–35 °C and has low affinity for both tight triple helix at 4 °C and gelatin at 42 °C, whereas MMP-3 prefers a tight triple helix at 4 °C and its affinity drops with decreasing triple-helicity at increasing temperatures (Fig. 4f). This agrees with our molecular modelling experiment (Figs. 2 and 3) which shows how snugly the triple helix fits into a valley running along the two domains of MMP-3. The putative collagen binding mode, represented by our complex 2, assumes that the main hydrophobic interaction occurs in the Cat domain, yet this domain alone is insufficient for collagen binding (Fig. 1a). This suggests that the Hpx domain plays an important role in positioning of the enzyme on the substrate. Similar cooperation between the domains is seen in MMP-1 interaction with the triple helix^22^, which shows a similar interface area between the binding partners (1290 A^2^ for MMP-1:THP vs 1141.7 Å^2^ for MMP-3:THP in complex 2). However, MMP-1 binding to the triple helix involves another hydrophobic (Leu) cluster, 9 residues away in the C-terminal direction from the Leu cluster that binds to the Cat domain. That second Leu cluster provides a strong anchor to the Hpx domain (*exosite*), which is predicted to be essential for triple helix unwinding^22,39^, the step required for collagenolysis^40^. There is no evidence of a similar mechanism for MMP-3, which we find to use a very compact recognition motif of a single triplet, GFO/A. This may be another reason why MMP-3 cannot unwind and cleave collagen^40^.

Our MMP-3:THP complex prediction results from incorporation of multiple lines of experimental evidence in the modelling process. Such a hybrid approach is increasingly applied to proteins and protein complexes that are resistant to crystallisation and too small for cryo-EM structure determination. So far there are no X-ray, NMR or cryo-EM structures of full-length MMP-3, let alone in complex with a THP, but this study provides key insights for a crystallisation trial of such a complex. Use of a relatively long THP, which spanned all the interaction sites with MMP-1 and extended well beyond the globular shape of the enzyme, proved beneficial for the crystal lattice formation in the case of the MMP-1:THP complex^22^. The same strategy could also lead to successful crystallisation of an MMP-3:THP complex, which is probably still too small (~50 kDa) for cryo-EM. Alternatively, microelectron diffraction (micro-ED) could be useful in this case, as it works even with very small ‘invisible’ crystals^41^.

**Figure 5:**
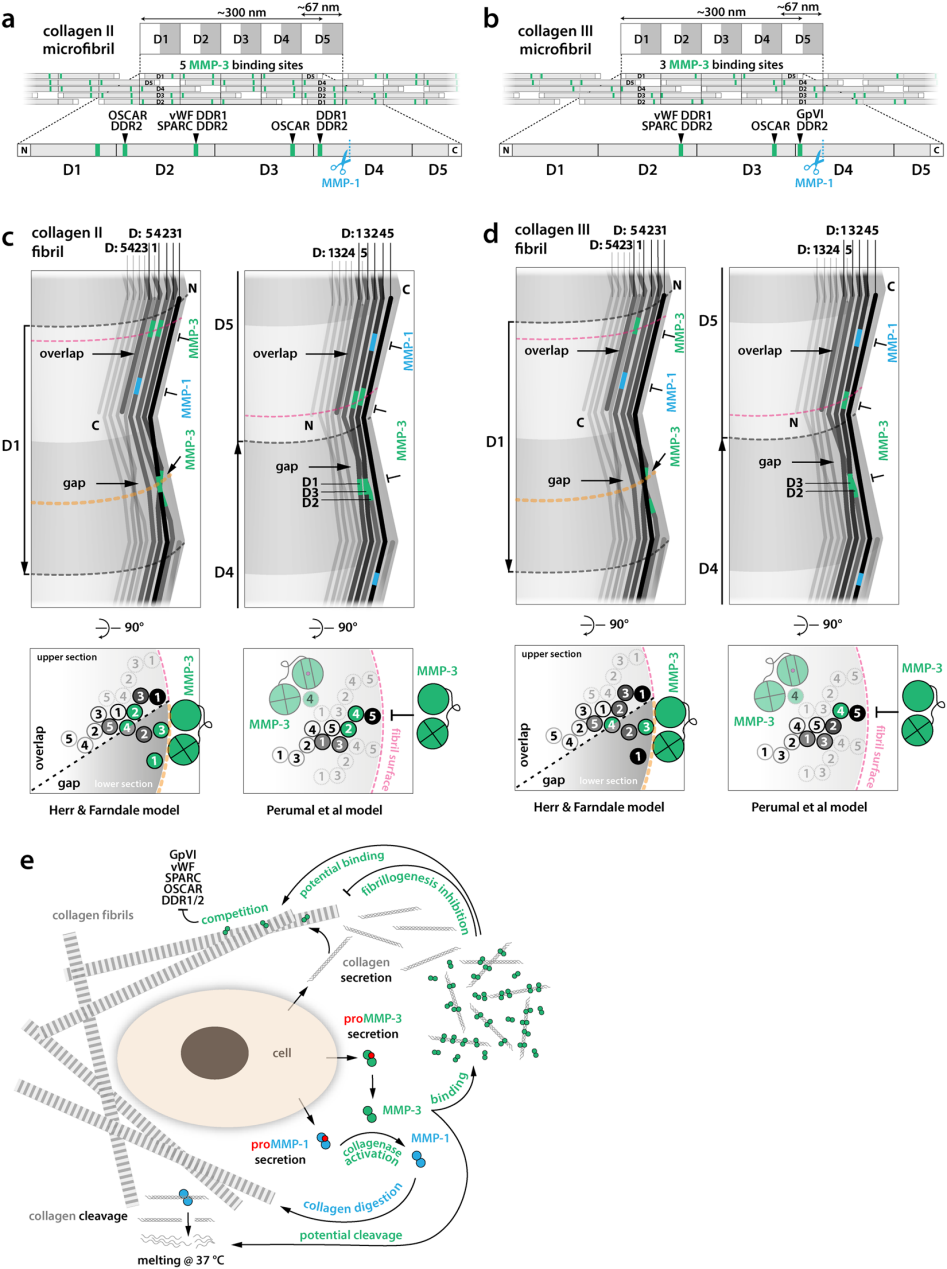
(**a**,**b**) Schematic microfibrillar organisation of collagen molecules forming gap and overlap regions (D-banding pattern). Each D-period contains the entire sequence of the collagen molecule distributed between five collagen molecules assembled with 1D stagger. Non-helical telopeptides on the collagen N- and C-termini are indicated with white boxes. MMP-3 binding sites overlap with the previously mapped sites for: discoidin domain receptors, DDR1 and DDR2 (GxRGQOGVMGFO); von Willebrand factor, vWF (GxRGQOGVMGFO); SPARC (secreted protein acidic and rich in cysteine) or BM-40/osteonectin; platelet activatory receptor Gp (glycoprotein) VI; and osteoclast-associated receptor, OSCAR (GxOGPxGFxGxO). (**c**,**d**) Schematics of the 3D arrangement of collagen microfibrils in the collagen fibril according to Perumal *et al*. and Herr & Farndale models and locations of MMP-3 binding sites (green) and MMP-1 cleavage sites (blue) in each arrangement. Collagen molecules are represented with zig-zag lines with grey levels fading towards the fibril centre. Lateral views (top panels) show interdigitation between collagen molecules in neighbouring microfibrils and their winding with respect to indentations on the surface of the collagen fibril, observed with scanning electron microscopy and atomic force microscopy. The Perumal *et al*. model assumes that the C-termini of collagen molecules are exposed on the surface of the fibril, whereas the Herr & Farndale model assumes that these are the N-termini. Fibrillar D-period labels are next to the arrows indicating fibril polarity (N → C) and are numbered according to the order of D-segments of the outermost, surface-exposed collagen molecules (black), corresponding to fibrillar D-numbering in A and B. The D-segment order of the individual collagen molecules is also indicated in the distinct staggered arrangements of each fibril model. Cross-section schematics (bottom panels) show selected collagen molecules, numbered according to their D-segment in the cross-section. All MMP-3 binding sites are buried in the idealised collagen fibril according to the Perumal *et al*. model, preventing MMP-3 access (T symbols). Only incomplete fibrillar assemblies would enable MMP-3 binding. The Herr & Farndale model predicts exposure of several MMP-3 binding sites on the fibril surface and their potential binding by MMP-3 (orange cross-sections). (**e**) Schematic illustration summarising potential functions of MMP-3 in the ECM.

Considering the biological significance of collagen binding by MMP-3, it is fascinating to discover that it can compete for binding sites with other fibrillar collagen ligands and thus may regulate their functions (Fig. 5a,b). These include the following proteins screened for Toolkit binding in the past, recently reviewed^25^: i) discoidin domain receptors (DDR1 and DDR2)^42^, which control mammary tissue (DDR1), long bones (DDR2), and are potentially involved in fibrotic states, atherosclerosis and cancer; ii) von Willebrand factor (vWF)^43^, essential for platelet adhesion to damaged blood vessel walls and in blood coagulation; iii) matricellular calcium binding protein SPARC (secreted protein acidic and rich in cysteine)^44^ also known as BM40 or osteonectin, which is counter-adhesive and anti-proliferative; iv) platelet activatory receptor glycoprotein GpVI^45^; and v) osteoclast-associated receptor (OSCAR), participating in osteoclastogenesis^46^ (Fig. 5a,b). In addition to the potential competition for the shared binding site on collagen II and III, MMP-3 can cleave SPARC into 3 biologically active peptides: Z-1, which increases angiogenesis, and Z-2 and Z-3, which inhibit cell proliferation^47^.

Schematic 2D models of collagen microfibrils, illustrating their characteristic D-periodicity, show clustering of the MMP-3 binding sites in both collagen II and III microfibrils (Fig. 5a,b). However, the actual arrangement of microfibrils in the collagen fibril is still unclear and the exposure or burial of MMP-3 binding sites – and those of many other collagen-binding proteins for that matter – may critically depend on this arrangement^48^. Two models of collagen microfibril packing in a collagen fibril have been proposed (Fig. 5c,d), based on an intermediate resolution structure of collagen fibril^49^: one model assumes exposure of tropocollagen C-termini on the fibril surface (Perumal *et al*.^50^), while the other assumes the exposure of the N-termini (Herr & Farndale^51^). Each model also results in distinct interdigitation between individual tropocollagens of the constituent microfibrils (Fig. 5c,d). Only the Herr & Farndale model predicts some of the MMP-3 binding sites be exposed on the idealised, intact surface of the fibril. The Perumal *et al*. model predicts all MMP-3 binding sites to be cryptic in such a fibril and MMP-3 could only bind to incomplete or damaged fibrils (Fig. 5c,d). Others suggest through molecular dynamics simulations that collagen fibrils are dynamic and ‘smart’, as they sample a collection of states in which cryptic sites are temporarily exposed or buried^52^. One of us has concluded that the evolution of binding sites for numerous ligands that are distributed across the tropocollagen molecule favours accessibility of collagen sequence that is not related to the D-period^25^. High-resolution fibre diffraction and/or correlative super-resolution light and electron microscopy (CLEM) may in the future use MMP-3, among other collagen binders, as a reporter protein to inform on the arrangement of collagen in the fibril.

In both models of collagen fibril assembly, a number of MMP-3 binding sites are concealed, consistent with the observed inhibition of fibrillogenesis in the presence of MMP-3 (Fig. 4g). This suggests that MMP-3 may regulate assembly of tropocollagens into higher order structures, as shown for SPARC^44^ that shares binding sites on these collagens with MMP-3. MMP-3(E200A)6 added at the ratio of 0.5:1 to collagen I, delayed fibrillogenesis by ~50 min, comparably to SPARC at a 10:1 molar excess over collagen^44^, which indicates that MMP-3 could be effective in blocking or regulating collagen assembly *in vivo*. The most striking pathological evidence of MMP-3 binding to collagenous matrix is in rheumatoid synovia^53^ or in colorectal cancer^54^. Such binding could indirectly enhance collagenolysis by rendering newly secreted collagen molecules more available to enzyme attack (Fig. 5e). It may also play a role in limiting collagen fibril diameter similarly to decorin^55^.

Both models of collagen fibril also assume that the collagenase cleavage site is concealed in the collagen fibril (Fig. 5c,d). According to the Perumal *et al*. model it is covered by the C-telopeptide of a neighbouring tropocollagen, whereas in the Herr & Farndale model it is buried deeper in the fibril. MMP-3, among other MMPs, has telopeptidase activity and could contribute to removal of the C-telopeptides, and thus facilitate collagenolysis by increasing the exposure of the collagenase cleavage site on the surface of the fibril. This would constitute another way in which MMP-3 could regulate collagenolysis *in vivo*.

The exposure or burial of any binding sites on collagens may be largely regulated by distinct fibrillar architectures that may not easily be reduced to any model. This includes hybridisation of multiple collagens (see further), leading to species more or less decorated with certain ligands and more or less proteinase resistant. Collagen fibrils in the body range in diameter from 10 to 500 nm^56,57^, depending on the specific tissue and its state of development. Collagen I is widespread, while collagen II is specific for cartilage^58^, and they both often co-distribute with Collagens V and XI, respectively^59–61^. The hybridisation of collagens II and XI leads to formation of ‘thin’ fibrils (16 nm)^62^, co-existing with homotypic ‘thick’ (40 nm) fibrils in the cartilage^63^. Collagen III is present in elastic tissues, mainly in blood vessels and skin and is often found at the periphery of type I collagen fibrils, especially in embryonic skin^64^. Some collagen fibrils become organised into elaborate hierarchical arrays of parallel rope-like fibres and bundles of fibres in tendons and ligaments, concentric layers in bones or orthogonal lattices in the cornea. In the latter, such an arrangement pertains to small diameter fibrils (~20 nm) and is essential for optical transparency^65^. This abundance of distinct hierarchical states of fibrillar collagens illustrates the need for tissue-specific mechanisms to regulate collagen fibril formation, where MMP-3 may play a role.

The observed multi-site collagen binding also suggests the potential mechanism of MMP-3 tissue retention. Decoration of interstitial collagen in the extracellular space may be a way to evade endocytosis through cell surface receptors. Several serine proteinase inhibitors, such as α_2_-Antiplasmin, α_1_-Proteinase inhibitor and α_1_-Antichymotryspin can be cleaved and inactivated by MMP-3^11^. This is important, as serine proteases like plasmin, plasma kallikrein or neutrophil elastase initiate proMMP-1 and proMMP-3 activation^66^. After such initial cleavage in the pro-domain, proMMP-3 autoactivates through autolysis and then completes activation of partially activated procollagenases. This complex network of reciprocal relations shows tight regulation of proteolytic activity, but on the other hand a potential for a positive feedback loop, where the enzyme escapes the inhibitory mechanisms. Such positive feedback may lead to rapid hyperactivation of MMP-3 along with many other MMPs, especially those downstream of MMP-3 in the activation cascade, such as the collagenases. Tissue destruction in numerous diseases can be attributed to such events. For example, aberrant collagenolysis is greatly manifested during joint destruction in rheumatoid and osteoarthritis. MMP-3 is upregulated post-myocardial infarction^12^ and in a mouse model, overexpression of MMP-1 caused functional heart defects due to a reduction in collagen^67^. MMP-1 is also one of several MMPs implicated in osteoporosis^68^, since collagen I degradation by MMP-1 initiates bone resorption by activating cathepsin K-producing osteoclasts^69,70^.

This poses an interesting question, whether the binding of the major procollagenase activator, MMP-3, to the collagen matrix may provide a reservoir of immediately bioavailable and uninhibited enzyme, ready to initiate a chain reaction when triggered by inflammation or a specific external factor. We showed that MMP-3 binding to tropocollagens I, II and III does not protect them against collagenolysis by MMP-1. According to our study, after collagen cleavage by MMP-1 or another collagenase, MMP-3 would be expected to lose affinity for the melting ¾ and ¼ fragments and dissociate from them. Once liberated from the collagen storage MMP-3 could boost the collagenolytic process by activating more procollagenases and by inactivating proteinase inhibitors, which could in turn lead to even more enhancement of MMP activation through serine proteinases. As previously outlined, MMP-3 could also aid collagenolysis by removing collagen telopeptides and by inhibiting fibrillogenesis of the newly secreted tropocollagens, increasing the collagenase access to the scissile bonds. Finally, it could also contribute directly to chopping of the pre-cleaved, unfolding collagen α-chains into smaller fragments (Fig. 4e). This essentially gelatinolytic activity did not appear potent in our hands and we think it is unlikely to be of great significance *in vivo*, considering the presence of proficient gelatinases, such as MMP-2 and −9, along with other proteases that can cleave unfolded collagen α-chains in the body. Nevertheless, MMP-3 treatment of *ex vivo* cartilage explants showed generation of several collagen II fragments^71^. Crucially, these fragments do not correlate with the MMP-3 binding sites mapped in this study, suggesting that MMP-3 binding to collagen II is unrelated to its gelatin cleavage specificity.

In summary, our work explains the mechanistic differences between a prototypic collagenase, MMP-1, and the non-collagenolytic stromelysin, MMP-3, encouraging a new perspective that closely related yet different MMPs can effectively act as stage-specific collagen chaperones, with collagenases capable of inducing and stabilising the unwound state of the collagen helix in order to subsequently cleave it, and stromelysins specialising in multi-site interactions with the tight triple-helical conformation to regulate collagen fibril assembly and, in turn, collagenolysis. It also defines a model, likely applicable to other extracellular enzymes, where a single enzyme can both directly and indirectly, and irrespective of its enzymatic activity, regulate aspects of ECM dynamics and activities of other extracellular proteins sharing common binding substrates. As such, the described functional potential of MMP-3 may be highly relevant in normal development, adult tissue homeostasis and diseases where dysregulated matrix turnover prevails.

## Methods

### Synthesis of collagen peptides (THP Toolkits)

Human collagen II and III THP Toolkits were synthesized by Fmoc (N-(9-fluorenyl)-methoxycarbonyl) chemistry as C-terminal amides on TentaGel R RAM resin using a CEM Liberty microwave-assisted automated peptide synthesizer and purified as described^72^. For exact sequences see references^42,72^. All peptides were verified by mass spectrometry and shown to adopt triple-helical conformation by polarimetry.

### Expression, refolding and purification of recombinant human MMP constructs

ProMMP-3, proMMP-3(E200A), proMMP-3Cat, MMP-3Hpx and proMMP-1 were generated according to the methods described by Chung *et al*.^40^. They were overexpressed from a pET3a vector in *E. coli* BL21 (DE3) strain (Invitrogen). Transformed cells were grown to OD600 ~ 0.4, then induced with 0.5 mM isopropyl-β-D-thiogalactopyranoside (IPTG), and harvested after 4 h. Inclusion bodies were collected by lysing the cells in 0.05 M Tris-HCl pH 8, 0.1 M NaCl, 1 mM ethylenediaminotetraacetic acid (EDTA), 0.26 mg/ml lysozyme (Sigma-Aldrich), 0.5% Triton-X100, and dissolved in 8 M Urea, 50 μM ZnCl_2_, 20 mM Tris-HCl, pH 8.6, 20 mM dithiothreitol (DTT). This was passed over a Macroprep HighQ ion-exchange column (BioRad), equilibrated in 8 M Urea, 50 μM ZnCl_2_, 20 mM Tris-HCl, pH 8.6, 1 mM DTT and eluted with a linear salt gradient (0–0.5 M NaCl). The fractions were run on SDS-PAGE and the relevant protein fractions were pooled, diluted with 50 mM Tris-HCl, pH 8.6, 6 M Urea, 1 mM DTT, 150 mM NaCl, 5 mM CaCl_2_, 100 μM ZnCl_2_, 0.02% NaN_3_ to A_280_ < 0.3, supplemented with 20 mM cystamine and refolded by dialysis at 4 °C against 4 volumes of renaturation buffer (50 mM Tris-HCl, pH 8.6, 150 mM NaCl, 5 mM CaCl_2_, 100 μM ZnCl_2_, 5 mM β-mercaptoethanol, 1 mM 2-hydroxyethyl disulphide, 0.02% NaN_3_) for 24 h, and then 10 volumes of the same buffer for another 24 h, then against 10 volumes of the same buffer without β-mercaptoethanol for 24 h, and finally against 4 volumes of 50 mM Tris-HCl, pH 8.6, 5 mM CaCl_2_, 50 μM ZnCl_2_, 0.02% NaN_3_, for 24 h. Refolded protein was purified using Green A affinity column (Amicon), equilibrated with 50 mM Tris-HCl pH 7.5, 75 mM NaCl, 5 mM CaCl_2_, 0.02% NaN_3_, and eluted with linear salt gradient (0–1 M NaCl). ProMMPs were activated with MMP-3Cat in 50:1 molar ratio and 1 mM p-aminophenyl mercuric acetate (APMA) (ICN Biochemicals) in TNC buffer (50 mM Tris-HCl pH 7.5, 150 mM NaCl, 10 mM CaCl_2_, 0.02% NaN_3_) for 60–120 min at 37 °C. The mature forms were purified by Sephacryl S200 gel filtration (GE Healthcare) in TNC buffer.

### Protein biotinylation

To avoid the presence of primary amines, the TNC buffer in which proteins were stored was exchanged using Sephadex G-25M PD-10 desalting gravity columns (GE Healthcare) into 50 mM N-Cyclohexyl-2-aminoethanesulfonic acid (CHES) buffer pH 8.8, supplemented with 200 mM NaCl and 10 mM CaCl2. Then, 10 mM EZ-Link Sulfo-NHS-LC-Biotin (Thermo Fisher Scientific) solution in distilled water was added at 1:2 protein-biotin molar ratio and incubated for 1 h at room temperature. Proteins were next passed over another PD-10 column equilibrated in TNC buffer to remove excess biotin.

### Acquisition of collagens I, II and III

Collagen I was extracted from Guinea pig dermis obtained from animal research facilities of Imperial College London. The skin was extensively scraped, cut into pieces, washed with saline and extracted with 0.5 M acetic acid. A part of the preparation was treated with pepsin for removal of the non-triple-helical telopeptides. Pepsin was added to 1/50 of the total wet weight for 24 h at 4 °C, and collagen was purified as described^73^. The pepsin-treated sample is less prone to fibrillogenesis. The non-pepsin-treated sample was stored as the fibrillogenesis-competent sample. Final collagen yield and concentration in both samples was determined after freeze-drying. Collagen II was a pepsin-digested guanidine-HCl-extract from a bovine joint cartilage purchased from Sigma-Aldrich/Merck. Bovine Collagen III was a gift from Dr. Shunji Hattori of Nippi Research Institute of Biomatrix in Toride, Ibaragi, Japan.

### THP Toolkits and collagen binding assays

For the Toolkits screening, Costar High Binding 96-well microtiter plates (Corning, UK) were coated with a 5 μg/ml THP solution in 10 mM acetic acid, incubated overnight at 4 °C. They were then washed with TNC buffer containing 0.05% Tween 20 (TNC-T) and blocked with 3% bovine serum albumin (Sigma) in TNC-T buffer. Biotinylated proteins at 1 μM concentration were added and incubated 1–2 h at room temperature. Plates were developed using streptavidin-horseradish peroxidase conjugate (R&D, UK) and 3,3′,5,5′- tetramethylbenzidine 2-Component Microwell Peroxidase Substrate Kit (KPL, UK) for a fixed time.

For collagen binding assay, the Costar plates were coated with 50 μl of 20 μg/ml collagen I, II or III in TNC buffer, incubated overnight at room temperature, washed and blocked as described above. Biotinylated proteins at increasing concentrations were added in TNC buffer and incubated for 2 h at 4–40 °C. The wells were then washed in TNC-T buffer at the temperature of incubation and subsequently fixed with 3% paraformaldehyde for 30 min. Plates were developed as in the Toolkits binding assay. All assays were carried out in triplicate and paired analyses were always developed simultaneously.

### Thermostability of collagens I, II and III and their MMP-1 cleavage products

Melting curves of collagens and their ¾ and ¼ fragments generated by collagenase were determined using circular dichroism (CD). Proteins were diluted to 10 μM concentration in TNC buffer and their thermal transitions were monitored by ellipticity (Θ) change at 222 nm across 0.1 cm pathlength in a Jasco 815 CD instrument. Temperature was increased at 0.1 °C/min.

### Collagenolysis assays

MMPs or trypsin were incubated with collagen in TNC buffer at indicated concentrations. Reactions were stopped at different time-points by the addition of an equal volume of the reducing ammediol loading mix [42 mM ammediol-HCl pH 7.5, 0.01% (w/v) NaN_3_, 2% (w/v), sodium dodecyl sulfate (SDS), 50% (w/v) glycerol, 1% β-mercaptoethanol and a few grains of bromophenol blue] containing 20 mM EDTA and 10 mM phenylmethanesulfonyl fluoride (PMSF). The cleavage products were analysed by SDS-PAGE using a modification of the ammediol-glycine gel and buffer system with 7.5% total acrylamide, and the gels were stained with Coomassie Brilliant Blue R-250.

### Turbidimetric collagen I fibrillogenesis assay

Collagen fibrillogenesis can be monitored by the extent of light scattering or turbidity. Fibrillogenesis-competent collagen I (non-pepsin-digested) at 1 μM was mixed with MMP-3(E200A) at the indicated concentrations in 20 mM Hepes pH 7.4, 150 mM NaCl, 2 mM CaCl_2_ in an Eppendorf UVette (220–1600 nm). The increasing sample turbidity was measured at 500 nm over for 4 h at 37 °C with a Cary 3 UV/VIS spectrophotometer equipped with a Varian temperature control unit.

### Homology modelling and docking

ProMMP-3 and mature MMP-3 models were obtained by homology modelling using Modeller 9.22 version^30^ via the web service using the default settings. The THP III-40d model for docking to MMP-3 model was obtained through replacement of residues within the 1BKV structure^26^ using Coot^74^ and UCSF Chimera^75^. The most common side chain conformations according to the Dunbrack rotamer library^76^ were selected for this starting model and then refined during Rosetta-assisted docking. The docking involved manual positioning of the THP model III-40d within a distance of 2–6 Å from the MMP-3 model in 4 indicated orientations, to decrease the global conformational search space and to improve the efficiency of the subsequent interface optimisation with Rosetta *relax* protocol^35^. The *relax* algorithm relieved clashes in the assembly and move it to the nearest local minimum in the Rosetta energy function.

### Structure visualisation and molecular interface analyses

Structural presentations were done using UCSF Chimera^75^. The interface areas in the modelled complexes were computed using NACCESS^36^. First, solvent accessible surfaces (SAS) of dissociated proteins were calculated by rolling a default probe (1.4Å size) around a van der Waals surface of each protein. Then the same operation was applied to the complexes and the interface areas were calculated as the difference between the two values. PDBePISA (http://www.ebi.ac.uk/msd-srv/prot_int/cgi-bin/piserver) was used to calculate theoretical free energy estimates of the modelled interfaces. ΔG^int^ is the solvation free energy gain upon interface formation. ΔG^int^ P-value indicates the interface specificity (P < 0.5 for specific interfaces). ΔG^diss^ is the free energy of dissociation, i.e. external energy needed to dissociate the complex.

## Supplementary information

Supplementary Information
