# Supplementary material for: Structural studies of the MMP-3 interaction with triple-helical collagen introduce new roles for the enzyme in tissue remodelling
Source: Sci Rep. 2019 Dec 11;9:18785. doi: 10.1038/s41598-019-55266-9 (PMC6906530; doi:10.1038/s41598-019-55266-9)
Supplement: Supplementary file 1 — Supplementary Information [file 41598_2019_55266_MOESM1_ESM.pdf]

Supplementary Information

Structural studies of MMP-3 interaction with triple-helical collagen introduce the enzyme's new roles in tissue remodelling

Szymon W. Manka[1†]*, Dominique Bihan[2] and Richard W. Farndale[2]

[1]Nuffield Department of Orthopaedics, Rheumatology and Musculoskeletal Sciences, Kennedy Institute of Rheumatology, University of Oxford, UK
[2]Department of Biochemistry, University of Cambridge, UK

*To whom correspondence should be addressed: s.manka@ucl.ac.uk
[†]Current address: MRC Prion Unit, UCL Institute of Prion Diseases, 33 Cleveland Street, London, W1W 7FF, UK

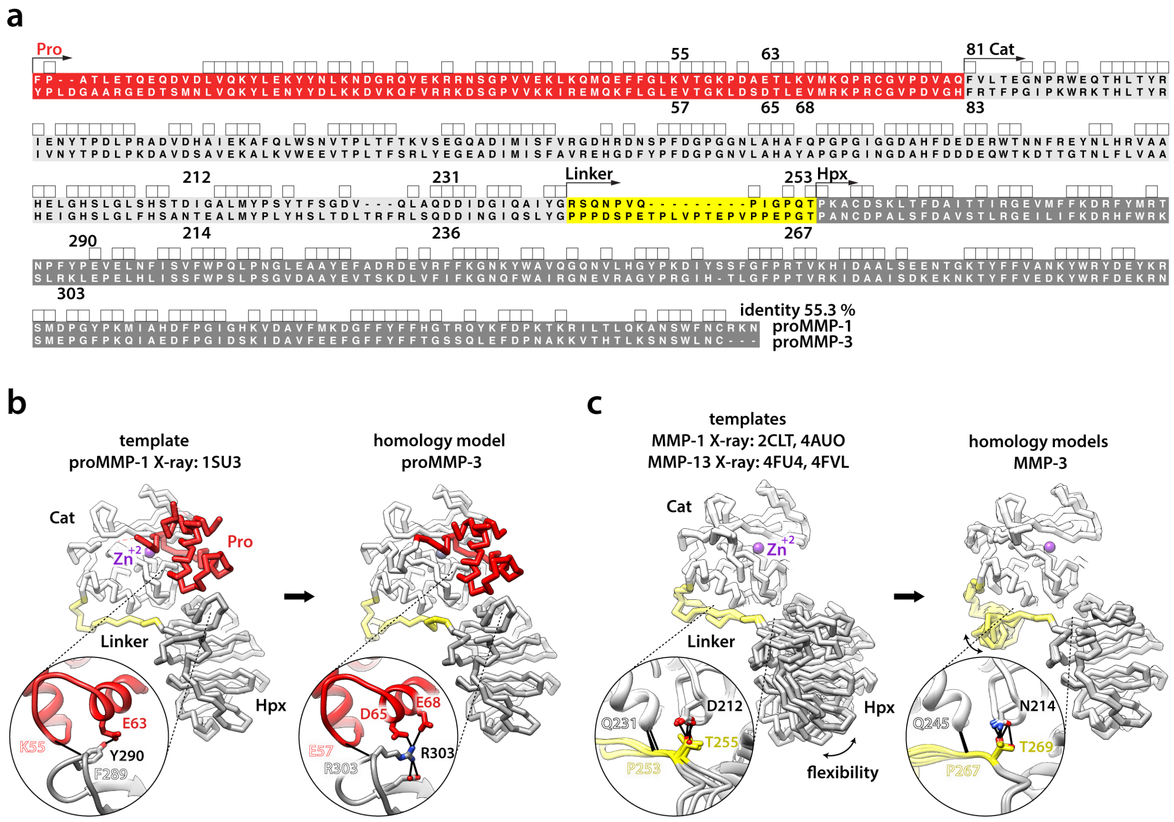

Figure S1. Homology modelling of full-length MMP-3 in the pro- and activated form. (**a**) Alignment of proMMP-1 and proMMP-3 sequences. Pro-domains, red; catalytic (Cat) domains, light grey; linkers, yellow; hemopexin domains (Hpx), dark grey. Selected, functionally important residues (see **b** and **c**) are numbered. Regions of 100 % conservation (sequence identity) are indicated with square symbols. MMP-3 linker is 9-residues shorter than that of MMP-1 (dashes). The overall sequence identity between proMMP-1 and -3 by Clustal X amounts to 55.3 %. (**b**) Homology modelling by Modeller of proMMP-3, based on the only available proMMP-1 crystal structure (PDB: 1SU3; backbone representations in the standard *frontal* orientation, facing the catalytic site cleft that contains the catalytic Zn^{2+} ion (magenta), with domain colouring as in **a**. Close-up views show the equivalent Pro-Hpx contacts (hydrogen bonds, black lines) contributing to the relative orientation of the Cat and Hpx domains in each proMMP structure/model; ribbon representation with selected residue side chains shown as sticks coloured by heteroatom (N, navy blue; O, red). Backbone contact labels, silhouette font; side-chain contact labels, solid font. (**c**) Homology modelling by Modeller of activated MMP-3, based on crystal structures of full-length MMP-1 (PDBs: 2CLT and 4AUO) and MMP-13 (PDBs: 4FU4, 4FVL). Shown are the 4 template structures and the 5 resulting MMP-3 models, superposed on their respective Cat domains; representation, view and colouring as in **b**. The superposition reveals inter-domain flexibility via linker (hinge region) in the activated MMP reference structures (templates) and a local conformational flexibility of the unstructured linker region in the target MMP-3 models, indicated by bent two-sided arrows. Close-up views show the equivalent linker-ordering regions along the Cat domain, focusing on selected relevant hydrogen bonds (black lines); ribbon representation with selected residue side chains shown as sticks coloured by heteroatom (N, navy blue; O, red). Backbone contact labels, silhouette font; side-chain contact labels, solid font. The

models show identical overall domain folds to the templates and the relative arrangement of the domains is preserved by analogous, however distinct interactions between them (see **b** and **c**, insets). Only the flexible and divergent linker, 9 aa longer in MMP-3 than in MMP-1 (see **a**), locally samples various conformations, where it is not ordered by the stabilising interactions with the Cat domain (see **c**).

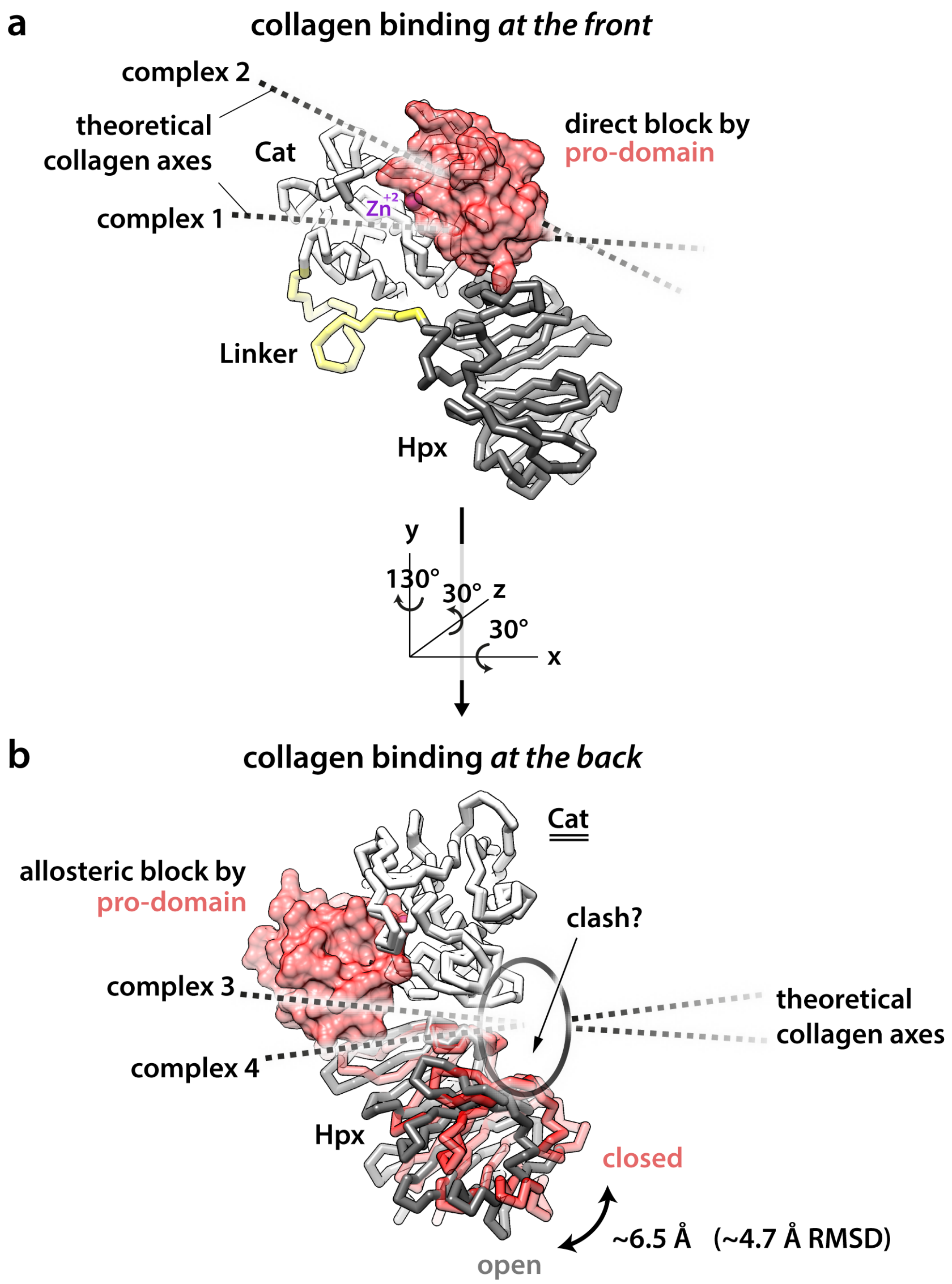

Figure S2. Models of direct and indirect collagen binding inhibition by the pro-domain of proMMP-3. (**a**) Direct steric hindrance would prevent collagen binding according to models 1 and 2**.** (**b**) Pro-domain-induced change in the relative positions of the Cat and Hpx domains could indirectly prevent collagen binding according to models 3 and 4. Homology models of proMMP-3 (red) and activated MMP-3 (grey) generated using proMMP-1 and MMP-1/13

templates (Figs. 2 and S1) are superimposed on the Cat domains (parallel lines) to demonstrate the potential widening of the cleft between the Cat and Hpx domains upon proMMP-3 activation. The closed conformation may be unable to accommodate collagen molecule in its binding site (clashing with the oval shape). Shown are maximal local displacement value and RMSD (root means square deviation) between the Hpx domains in the two models.

	complex 1	complex 2	complex 3	complex 4	THP	
Rosetta total score	-744	-742	-743	-735	**1BKV**	**III-40d**
Clash score	1.99 (99th percentile)	1.56 (99th percentile)	1.70 (99th percentile)	2.41 (99th percentile)	10.68	7.33
Poor rotamers	0	0	0	0	0	0
Favoured rotamers	99.7 %	99.7 %	99.7 %	98.6 %	84.4 %	88.5 %
MolProbity score	1.28 (98th percentile)	1.36 (98th percentile)	1.36 (98th percentile)	1.40 (97th percentile)	1.55	1.40

Table S1. Model refinement statistics table. Rosetta total score combines multiple terms of the Rosetta energy function and is expressed in arbitrary units: lowest scores correspond to most energetically favourable complexes. Clashscore, rotamers and MolProbity scores are determined using the MolProbity[1] server. MolProbity score combines the clashscore, rotamer, and Ramachandran evaluations into a single score. For comparison, included are the relevant scores of the THP structure solved by X-ray diffraction (PDB: 1BKV) and those of the III-40d THP built on the 1BKV backbone. Rosetta refinement has reduced internal clashes and improved rotamer conformations of III-40d in the complex with MMP-3, resulting in excellent overall stereochemistry in all four complexes (>97th percentile among N=27675 structures of 0-99 Å resolution).

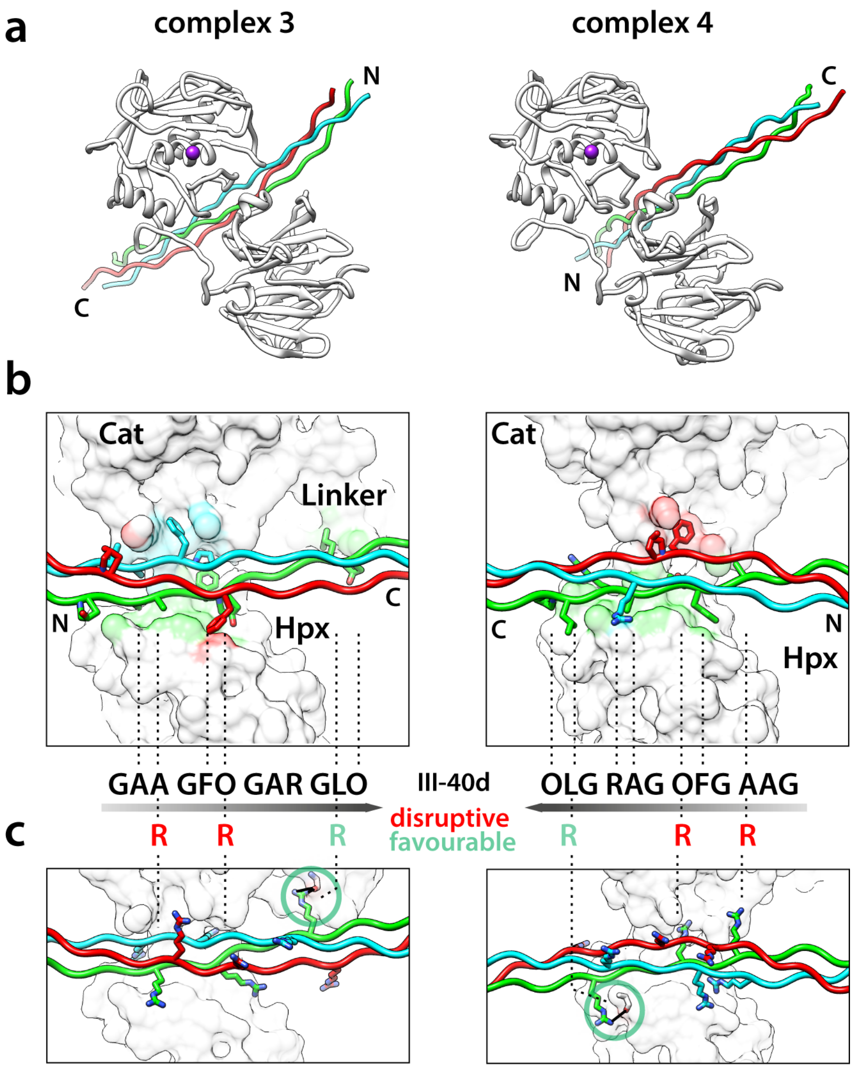

Figure S3. Modelling of the effects of collagen sequence variations on collagen binding to the back of MMP-3. (**a**) Two modelled back-binding MMP-3:III-40d complexes shown as ribbons in the standard MMP-3 orientation. MMP-3 chain, white; Zn^{2+}, magenta. THP chains: leading, blue; middle, green; trailing, red. (**b**) Close-up view on the modelled MMP-3:III-40d interfaces. THP representation as in **a**, with side chains of the residues containing at least one atom located within 4 Å distance from MMP-3 shown as sticks coloured by heteroatom: N, navy blue; O, red. MMP-3 is shown as solvent-accessible surface (SAS) (UCSF Chimera[2]) coloured according to the interfacing THP chains (4 Å distance). IDs of the staggered residues in the III-40d THP are roughly indicated (dashed lines) together with the THP polarity (shaded arrows). (**d**) Theoretical analysis of the effects of III-40d residue substitutions on MMP-3 binding. MMP-3 shown as white and transparent SAS and III-40d shown as in **a**, with modelled Arg side chains and hydrogen-bonded MMP-3 residues represented with sticks coloured by heteroatom: N, navy blue; O, red. Arg at the position X of the Triplet 2 forms additional hydrogen bonds (black lines) with MMP-3 in both models (green circles). Arg at the position Y of the Triplets -1 and 0 show no obvious effect on MMP-3 binding.

Supplementary references

1. Williams, C. J. *et al.* MolProbity: More and better reference data for improved all-atom structure validation: PROTEIN SCIENCE.ORG. *Protein Sci.* **27**, 293–315 (2018).
2. Pettersen, E. F. *et al.* UCSF Chimera--a visualization system for exploratory research and analysis. *J. Comput. Chem.* **25**, 1605–1612 (2004).
